# SHIFT: Server for hidden stops analysis in frame-shifted translation

**DOI:** 10.1186/1756-0500-6-68

**Published:** 2013-02-23

**Authors:** Arun Gupta, Tiratha Raj Singh

**Affiliations:** 1 School of Computer Science and IT, DAVV, Indore, M.P., India; 2 Department of Biotechnology and Bioinformatics, Jaypee University of Information Technology (JUIT), Waknaghat, Solan, H.P., India

**Keywords:** Frameshift, Reading frames, Hidden stop codons, Codon usage

## Abstract

**Background:**

Frameshift is one of the three classes of recoding. Frame-shifts lead to waste of energy, resources and activity of the biosynthetic machinery. In addition, some peptides synthesized after frame-shifts are probably cytotoxic which serve as plausible cause for innumerable number of diseases and disorders such as muscular dystrophies, lysosomal storage disorders, and cancer. Hidden stop codons occur naturally in coding sequences among all organisms. These codons are associated with the early termination of translation for incorrect reading frame selection and help to reduce the metabolic cost related to the frameshift events. Researchers have identified several consequences of hidden stop codons and their association with myriad disorders. However the wealth of information available is speckled and not effortlessly acquiescent to data-mining. To reduce this gap, this work describes an algorithmic web based tool to study hidden stops in frameshifted translation for all the lineages through respective genetic code systems.

**Findings:**

This paper describes SHIFT, an algorithmic web application tool that provides a user-friendly interface for identifying and analyzing hidden stops in frameshifted translation of genomic sequences for all available genetic code systems. We have calculated the correlation between codon usage frequencies and the plausible contribution of codons towards hidden stops in an off-frame context. Markovian chains of various order have been used to model hidden stops in frameshifted peptides and their evolutionary association with naturally occurring hidden stops. In order to obtain reliable and persuasive estimates for the naturally occurring and predicted hidden stops statistical measures have been implemented.

**Conclusions:**

This paper presented SHIFT, an algorithmic tool that allows user-friendly exploration, analysis, and visualization of hidden stop codons in frameshifted translations. It is expected that this web based tool would serve as a useful complement for analyzing hidden stop codons in all available genetic code systems. SHIFT is freely available for academic and research purpose at http://www.nuccore.org/shift/.

## Findings

### Background

Reading frames play an important role in the process of translation of nucleotide sequences into proteins. Selection of a wrong reading frame may alter the protein product. Such events that alter the reading frame occur extremely rarely during translation; Frame-shift is one such event. Frame-shifting is one of the three classes of recoding of mRNAs. Recoding is the reprogramming of mRNA translation by localized alterations in standard translation rules [1,2]. Frame-shift is quite common in viruses and also occurs in bacteria, yeast and other organisms [3]. It is a type of genetic mutation caused generally by indels, i.e. insertion and deletion of nucleotides. Coding sequences lack stop codons but myriad of stop codons materialize off-frame. Off-frame stops i.e. stop codons in +1 and −1 shifted reading frames, are termed as hidden stop codons or hidden stops (HSCs). Frame-shifts lead to the waste of energy, resources and activity of the biosynthetic machinery. In addition, some peptides synthesized after frame-shifts are probably cytotoxic which serve as plausible cause for innumerable number of diseases and disorders such as muscular dystrophies, lysosomal storage disorders, and cancer [1,2,4-6]. Frame-shift mutations might be beneficial sometime such as a frame-shift mutation was responsible for the creation of Nylonaser [7].

Here we present a user-friendly web based algorithmic application named SHIFT, to predict HSCs in coding genomic sequences. The inference methodology is based on a naïve sliding window as well as a stochastic approach. Later approach has been applied by utilizing Markov chains of various orders to generate Hidden Markov Models (HMMs) to predict HSCs by modulating natural coding sequences in to predicted ones and then analyzing results comparatively. A variety of features such as half gene analysis and evolutionary analyses, which includes relative synonymous codon usage (RSCU), relative adaptiveness and codon adaptive index (CAI), were incorporated to analyze the data to infer HSCs and their influence on putative functional, biochemical, and evolutionary events.

### Implementation

Here, we developed a web based application (SHIFT) that deals with a putative mechanism of frameshift which reflects the selection pressure acting on the production of off-frame translation products. SHIFT aims to identify the hidden stops in genomic coding sequences in both +1 and −1 frame-shift with respective genetic code systems. It classifies codon usage frequencies according to the contribution of codons towards hidden stops and their respective genetic code systems. Further it calculates the correlation between codon usage frequencies (CUF) and contribution of codons to hidden stops in off-frame context in the provided coding sequence(s), according to Seligmann and Pollock [4]. Additionally, one tailed t-test is also performed to generate the t-values for statistically significant correlations.

Underrepresentation and overrepresentations of nucleotides might be analyzed as mono, bi, tri, tetra, and so on, while tri is more common and useful because of triplet nature of codons and their significance in genetic code systems. It has been shown that the frequency of utilization of each codon in various organisms is directly proportional to the intracellular concentration of transfer RNA (tRNA) that decodes it. This adaptation optimizes the protein translation process by exactly adjusting the tRNA demand of the translation machinery to the amount available in the cell. The statistical biases may be used for purposes of prediction in answering questions of biological importance associated with DNA coding region, number of tRNA molecules tried, coding phase, and expression levels of genes [8,9].

The most powerful method for such determinations relies on a priori hypotheses, consists in seeking period 3 irregularities in nucleotide distribution. Non-uniform codon usage within a gene or exon should be revealed by period 3 bias in the frequencies of occurrence of individual nucleotides. The frequency of occurrence of each base at positions *3n*, *3n* + *1*, and *3n* + *2* are simply calculated and compared with the average frequency of occurrence in the sequence. The frequency of occurrence *f*_*1*_ of *N* codons in a window on phase 1 is compared with the individual frequencies of codons in the standard as well as other applicable genetic code usage table.

$$f_{1}=\prod_{i=1}^{N}f_{\text{codoni}}$$

By shifting first one, and then two nucleotides, the calculation is repeated for phases 2 and 3, which gives two other frequencies, *f*_*2*_ and *f*_*3*_. The probability of each phase being the coding phase is calculated by Bayes’ formula:

p1=f1f1+f2+f3

p2=f2f1+f2+f3

p3=f3f1+f2+f3

By displacing the length *N* window along the sequence, it is possible to trace the profiles of probabilities *p*_*1*_, *p*_*2*_, and *p*_*3*_, whose peaks indicate the positions of genes that code for proteins with remarkable precision. To analyze probabilistic profiles we have implemented HMMs through Markov chains of various orders (0 to 2), to model uni, bi and tri positional nature of nucleotides for frameshift (uni, and bi) and codon usage (tri).

The expression level of a gene may be estimated by comparing its codon usage frequency with the standard codon frequencies given in the codon usage table for the species. The gene expression level may be quantified using CAI, which is calculated as follows: Each codon *i* contained in the gene is assigned a score *w*_*i*_ equal to the ratio of its frequency to the frequency of the most frequent codon that codes for the same amino acid. If codon *i* is the most frequent, then *w*_*i*_ equals 1. For a codon that is systematically avoided, *w*_*i*_ is close to 0. The CAI is the geometric mean of the *w*_*i*_ scores of the set *L* of the gene’s codons [8-10].

(5) $$\text{Index}={\left(\prod_{i=1}^{L}w_{i}\right)}^{\frac{1}{L}}$$

The CAI score obtained ranges between 0 and 1, and increases as the gene conforms to the standard utilization frequency of the species’ genetic code. For example, protein genes that are strongly expressed in the yeast, such as ribosomal proteins and histones, have scores of between 0.52 and 0.92, whereas regulatory protein genes, of which there are only a few copies per cell, have a score of 0.1. The CAI may be used to estimate the expression level of a gene whose function is unknown. It is also useful when expressing a recombinant protein in a heterologous host, for example, a human protein in a bacterium. The CAI for a human gene, in combination with the bacterial codon usage table, allows us to predict whether a gene will be efficiently expressed, and may be used to guide the modification of certain codons in order to better adapt the gene to its new host. Analysis involving CAI and CUF will definitely furnish new insight to the codon usage and their functional implications [10].

**Figure 1:**
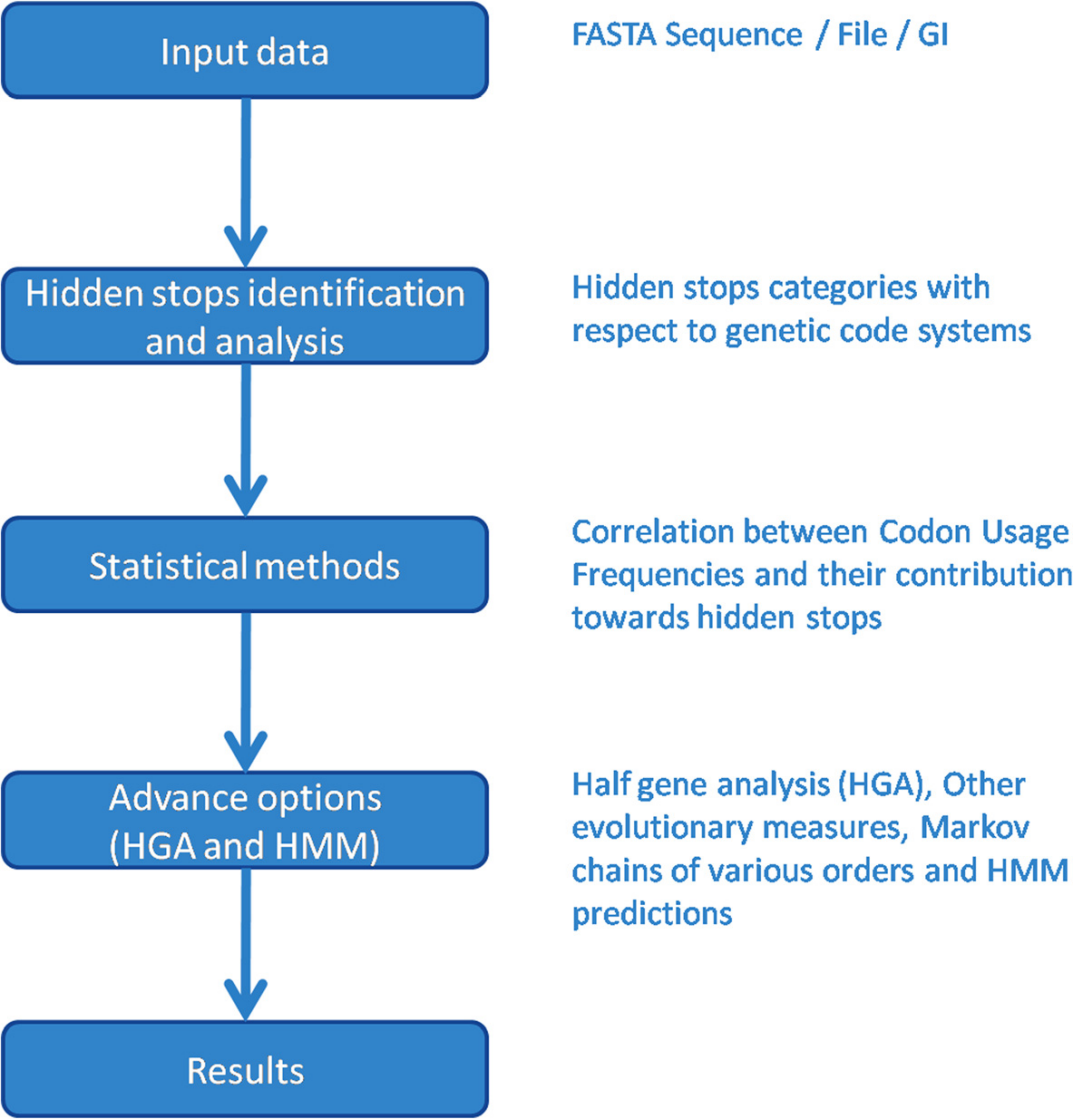
Schematic illustration of SHIFT with all technical details.

SHIFT is written in Perl and uses a web interface developed with CGI. Multiple scripts are used to produce output from the given input data (Figure 1). We have implemented GD package from Perl repository to generate the correlation graphs. Email package has been used to send the link for the stored results to the email id provided by user. The SHIFT server is tested with myriad of available nuclear as well as mitochondrial genomic sequences according to respective genetic code systems with different sizes and number of input sequences.

### Usage

The main interface of the SHIFT is a graphical display with all options and menu available in one screenshot. SHIFT accepts as input one or more coding DNA sequence(s). Coding DNA sequence(s) can be typed/pasted in the given text box or can be provided as a file input in FASTA format. GeneIDs alone (GI, Genbank, NCBI) may also be provided as an input. Desired genetic code can be selected from the given list of available genetic code systems. Necessary information wherever required is provided online (in Overview and FAQs sections), that describes all the operations and methodology implemented. A comprehensive result will be displayed to the user in tabular as well as graphical form. Results generated will be stored on the server for a period of one month and a link to these results would be sent via email to the user if opted.

### Results and discussion

There is general agreement that codons are translated at different rates [11]. The first indication of non-uniform translation rates was the observation that there are pauses during polypeptide elongation and that these can be identified with short strings of rarely used codons [12,13]. It became an accepted opinion that biased codon usage could regulate the expression levels of individual genes by modulating the rates of polypeptide formation [14,15]. With the sequencing of significant number of genes and genomes, there are several occurrences of codon reassignments, premature stop codons, and read through stop codons in protein coding sequences at various taxonomic levels [16-18]. There are computational evidences for phylogenomic selection of hidden stops [19].

One of the most prominent fundamental issues in biology that remain unresolved is that of the evolution of gene expression. It has been suggested that the complexity and evolution of gene expression and the noise in gene regulation and expression is hard to understand because of the divergent expression patterns [20]. There are several evidences where codon usage bias have been correlated positively with the expression levels of genes; in *E*. *coli*[19,21,22], in *Saccharomyces cerevisiae*[23], and in nitrogen fixing endosymbiont *Bradorhizobium japonicum*[24]. Evidences for positive correlation between codon usage bias and gene’s size are also shown in *Drosophila*[25], in *E*. *coli*, *Arabidopsis*, *Holobacterium* and *Homo*[26], and in yeast [27].

Different rate of protein evolution is a central problem in molecular evolution. The best predictor of evolutionary rate is expression level. Pressure for translational sturdiness increases with expression level and restrains sequence evolution [28]. For large and high expression level genes, the cost of off-frame translation is likely to increase. Several hypotheses like functional loss, translational efficiency, and translational robustness implies that selection can act on nucleotide sequence, to increase the translational accuracy by optimizing codon usage, and on amino acid sequence, to increase the number of proteins that fold properly [29,30]. It is estimated that this correlation might be reflected in first half of coding sequences and suggested that hidden stops should be more frequent in the first half of the gene [4,19].

It was implied that at the level of genetic codes, ancient adaptive events may have adjusted codon assignments to increase frequencies of codons that can be part of hidden stop codons [4]. Assuming that hidden stop codons are uncorrelated with the average codon usage frequencies (*null hypothesis*), we expect 0.05 of the organisms tested to show significant correlation between hidden stop codon and codon usage frequencies [4,19]. Thus, to test whether the *null hypothesis* holds and to confirm the statistically significant correlation, t test (P < 0.05, one-tailed test) was implemented along with correlation in SHIFT.

To identify and evaluate the preferable positions of hidden stops in coding sequence(s) we implemented half gene analysis in SHIFT. To implement this, hidden stops in both +1 and −1 frame-shifts are identified by dividing gene into codons and then divide it into two equal parts. Tabular results for half gene analysis will be reported to the user. GC bias is the main determinant of hidden stops frequencies in coding sequences. Similar kind of studies has also been proposed about G and C contents of genomic coding sequences [31-33]. To evaluate this parameter for multiple (5 or more) coding sequences, SHIFT calculates correlation between G + C content and frequency of hidden stops in coding sequences (see supporting data online).

There are evidences where frameshift events are being utilized to analyze coding sequences to interpret biologically meaningful results. Evidences of phylogenetic trends in analyzing frameshift events in various lineages were reported [19]. Study on associations between developmental stability and hidden stops favor an adaptationist interpretation as structuring the genetic code and its evolution [34]. Another interesting study on frameshift and its implications in prokaryotes suggested and support the hypothesis that OSCs (out of frame stop codons) carry functional significance and have been selected in the course of genome evolution to act against unintended frameshift occurrences. Some results also hint that OSC overrepresentation being a compensatory mechanism to make up for the decrease in OSCs in high G + C organisms, thus revealing the interplay between two different determinants of OSC frequency [35].

Importance of this putative event could be reflected in some recent studies based on frameshift mutations and their involvement in various diseases and other biological machineries such as diamond-blackfan anemia [36], E. coli’s association with chromosomal reference and mutational sites [37], Retinitis punctata albescens [38], Pendred syndrome in Korean population [39], and microsatellite instable gastric and colorectal cancers [40]. Therefore SHIFT will help molecular and evolutionary biologists to verify various aspects related to this putative evolutionary event of frameshift mutations and will provide new directions to the research in this area. It also provides opportunities to discuss other evolutionary events and to associate them with this mechanism. It is hoped that this web based tool would serve as a useful complement for analyzing hidden stop codons in all the lineages through their respective genetic code systems. Additionally it will help to manipulate the biological sequences through theoretical modeling of natural biological sequences by applying Markov chains of various orders through HMMs and to analyze its impact on natural sequences and their future biological predictions.

## Availability and requirements

· Project name: SHIFT – Server for Hidden stops analysis In Frame-shifted Translations

· Project home page: http://www.nuccore.org/shift

· Programming language: Perl/CGI

· Other requirements: Web enabled services from standard web browsers

### Availability of supporting data

The Results of this article and their brief interpretations obtained from SHIFT on real genomic data are available in the [supp_info] repository, [http://www.nuccore.org/shift/supp_info].

## Abbreviations

HSCs: Hidden stop codons or hidden stops; HMMs: Hidden Markov Models; RSCU: Relative Synonymous Codon Usage; CAI: Codon Adaptive Index; CUF: Codon Usage Frequencies; OSCs: Out of frame Stop Codons.

## Competing interests

The authors declare that they have no competing interests.

## Authors’ contributions

TRS conceived the problem. AG wrote the initial version of SHIFT under the supervision of TRS. Subsequent versions were prepared under the supervision of TRS. AG wrote the code and developed web enabled services for SHIFT under the supervision of TRS. AG and TRS wrote the manuscript. Both the authors read and approved the final manuscript.
